# Polymer Brushes under High Load

**DOI:** 10.1371/journal.pone.0058392

**Published:** 2013-03-13

**Authors:** Suzanne M. Balko, Torsten Kreer, Philip J. Costanzo, Tim E. Patten, Albert Johner, Tonya L. Kuhl, Carlos M. Marques

**Affiliations:** 1 Department of Chemical Engineering and Materials Science, University of California Davis, Davis, California, United States of America; 2 Leibniz-Institut für Polymerforschung Dresden e.V., Dresden, Germany; 3 Department of Chemistry, University of California Davis, Davis, California, United States of America; 4 Institut Charles Sadron, Université de Strasbourg, CNRS, Strasbourg, France; Harbin Institute of Technology, China

## Abstract

Polymer coatings are frequently used to provide repulsive forces between surfaces in solution. After 25 years of design and study, a quantitative model to explain and predict repulsion under strong compression is still lacking. Here, we combine experiments, simulations, and theory to study polymer coatings under high loads and demonstrate a validated model for the repulsive forces, proposing that this universal behavior can be predicted from the polymer solution properties.

## Introduction

Nature and formulation scientists have chosen high molecular weight polymers as the most effective coatings to achieve large repulsive forces between surfaces. Such strong repulsions are required in lubrication [1]–[3] and play an important role in colloidal stabilization [4]. End-grafted polymer layers are, in this context, prominent model systems. But despite two decades of extensive work on such so-called polymer brushes via experiments [5]–[11], theory [12]–[18], and computer simulations [19]–[23], a validated, quantitative model to predict the repulsive forces generated by these soft coatings under high compressions is still lacking [11].

The polymer brushes used in our physical experiments were composed of polyethylene glycol (PEG) - polystyrene (PS) diblock copolymer chains grafted to mica in toluene, a selective solvent for the PS block [5], [6]. Two samples of moderately polydisperse PEG-PS diblock copolymers were studied with equal number-average molecular weight, M_n_ = 5,000 g mole^−1^, of the PEG anchor block and two different PS blocks. In addition, we measured force profiles of a similar PEG-PS diblock polymer with low polydispersity. Molecular weight, polydispersity (PDI = M_w_/M_n_), grafting density, and stretching factor, *C*, of the three systems are indicated in Table 1. The synthesis and characterization of the polymers is presented elsewhere [24].

**Table 1:** Physical properties of the polymers under investigation.

	PS		PEG		PDI	Grafting Density	
Polymer	N	M_n_	N	M_n_	M_w_/M_n_	*σ* chains/nm^2^	*C*
PS37K	355	37 k	114	5.0 k	1.23	0.195	3.5
PS37k**	355	37 k	148	6.5 k	1.06	0.193	3.5
PS88k	845	88 k	114	5.0 k	1.34	0.013	2.1
Simulation	60	N/A	N/A	N/A	1.23	0.091	3.5

The grafting density, *σ*, of each system was determined via quartz crystal microbalance (QCM-D) onto quartz substrates. A value of 1.0 or greater in the stretching factor, 
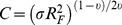
, of the polymer brush indicates a strongly stretched brush [20] with 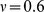 and *R_F_* the Flory radius. **Copolymer was obtained from Polymer Source (Quebec, Canada) and does not contain thiol functionalization.

In the present work, we present a comprehensive analysis of polymer brush repulsion, including the high compression regime. The polymer brushes in this work were studied using the Surface Force Apparatus (SFA), as previously introduced [25], [26]. To gain insight into the cause of deviations in the force profile under high compression, we compare experimentally measured force profiles of opposing polymer brushes as a function of distance to molecular dynamics (MD) simulations. From this quantitative comparison, we propose modifications to mean-field theory, which include high volume fraction effects that accurately account for deviations from ideality in the constitutive equation of state for bulk polymer solutions. As we will demonstrate, the modified theory accurately predicts the simulation and experimental data, hence reiterating that polymer osmotic properties are the basic design principle of strongly repulsive polymer coatings.

## Materials and Methods

### Polymer Synthesis

End functionalized diblock copolymer molecules were synthesized with a constant PEG block of 5,000 g · mole^−1^ and varied molecular weight PS block containing a thiol (SH) moiety attached to the terminus of the PS block. Three specific copolymers were used in this work, PEG6.5k-PS37k (purchased from Polymer Source, Quebec, Canada), PEG5k-PS37k-SH, and PEG5k-PS88k-SH. Details of their properties are given in Table 1. The thiolated polymers were synthesized via RAFT polymerization and the non-thiolated polymer from Polymer Source was synthesized by a living anionic polymerization. The detailed synthesis and characterization of the thiolated polymers listed in Table 1 is presented elsewhere [24].

### Surface Force Apparatus

The Surface Force Apparatus (SFA) has been described previously and used extensively to measure the interactions between various surfaces [25]–[27]. Here, we describe the specific apparatus properties for our experiments. The sample surfaces were glued down to cylindrically curved supports using a 1∶1 molar ratio of dextrose-*D*-galactose sugar mixture. The silver surface was produced via thermal evaporation of pure, 99.999%, silver onto freshly cleaved mica substrates. After preparation, the surfaces were immediately mounted into a Mark II SFA chamber. The top surface was fixed and the lower surface was mounted onto a double cantilever spring of spring constant 4.6×10^5^ mN · m^−1^. At the beginning of each experiment, two bare mica surfaces were brought into contact in air to ensure that the surfaces were clean, to measure the zero contact position and determine the mica thickness. Afterwards, the surfaces were separated ∼1 mm and an excess of polymer solution was introduced between the surfaces to ensure saturation of the surfaces with polymer. The surfaces were then allowed to incubate with the polymer solution between the surfaces for a minimum of 8 hours before the experiment was continued. After incubation, the SFA chamber was filled with spectral grade toluene. First, measurements were performed on the double brush system. Several contact positions and a minimum of 3 approach and separation cycles were performed on each position to ensure no hysteresis was present. Next, the top polymer-covered surface was replaced by a bare, back silvered mica substrate and single brush measurements were performed in the same manner as the double brush measurements. Surface separations were determined to an accuracy of ±0.5 Å and intersurface forces were determined to an accuracy of ±4% [28]. The average time for one single approach and separation was approximately 1 hour. Experiments were performed with the surface separation controlled both manually and automatically by our own in-house software. In the case of automated control, FECO images were acquired using a 2,048 pixel×512 pixel CCD detector (Princeton Instruments) with a resolution of ±0.25 Å in wavelength and ±1 µm lateral distance across the sample surface. FECO peak wavelengths were identified to ±0.1 Å as previously described [28].

### MD Computer Simulations

Our experiments are compared to molecular dynamics (MD) simulations of a classical, coarse grained polymer (Kremer-Grest) model [23]. Monomers are represented by excluded volume (Lennard-Jones) spheres, interacting via a Lennard-Jones potential [23],
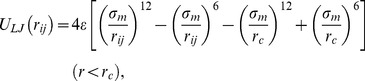
(1)


where *ε* and *σ_m_*, respectively, define the units of energy and length. The distance between monomer i and j is denoted by *r_ij_* and *r_c_* is the cut-off radius of the potential. Choosing 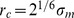 at the minimum of the Lennard-Jones potential restricts the excluded volume interaction to be purely repulsive. This ensures good solvent conditions of our (athermal) model, although we apply an implicit treatment of the solvent. *U_LJ_* is not only truncated but also shifted to avoid a discontinuous force at *r_c_*.

The connectivity between adjacent monomers of a chain is realized via the FENE potential [23],
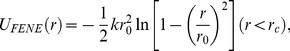
(2)


where r is the distance between monomers, 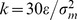 the spring constant, and 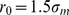 the maximum allowed bond length.

Our polymers are grafted with one chain end onto a rigid, hexagonal lattice with total area 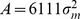. To this extent, we use the same potential as in Eqn. 1, but make the interaction between the surface and the end-monomer strongly attractive by doubling of *r_c_* and increasing *ε* by a factor of 100. All other monomers interact purely repulsive with the surface via the Lennard-Jones potential.

We solve the classical equations of motion using the Velocity-Verlet algorithm [29] with a time-step of 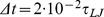, where 
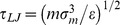
 is the Lennard-Jones time unit. For all monomers, the mass *m* is set to unity.

Temperature is kept constant at 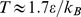, where *k_B_* is the Boltzmann constant, via the Dissipative Particle Dynamics thermostat. The latter is described in detail elsewhere [29]–[32]. Our simulation model is well established and has been used in a variety of studies concerning the properties of polymer brushes [21]–[23].

The average chain length, 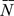, is 60 with a polydispersity of 1.23. In the setup of the simulation, the appropriate number of polymer chains are chosen at random from a Flory-Schulz distribution and grafted onto the surface. By randomly choosing both a grafting site and a polymer length from a Flory-Schulz distribution, we assure that there is no correlation between grafting sites and chain length.

### Polydispersity

As can be seen in Table 1, it will be necessary to consider the polydispersity of the polymers when comparing to mean field theory. Here, the polydispersity of the chains is represented by a Flory-Schulz chain length distribution 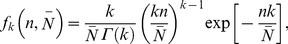
(3)


where 
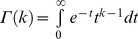
 and 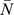 the average chain length. A Flory-Schulz distribution is known to describe well both the higher polydispersity, copolymers synthesized by RAFT polymerization [24], and the lower polydispersity copolymer made by living anionic polymerization [33] (Polymer Source, Quebec, Canada). The polydispersity is given by the parameter *k*, where PDI = 1+1/*k*. Note that the surface density of end-grafted chains with more than *n* monomers is given by 
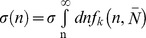
.

### Data Normalization

To compare the experimental and simulation data, it is logical to use the results of the classical MWC theory [1], [14] to normalize the data. First, we note that mass conservation between the two opposing surfaces requires that 
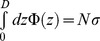
, with Φ(z) the monomer density, *N* the polymerization index, and σ the end-grafted surface density. In the mean-field approximation, the pressure *p* is 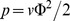 and the potential 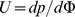, derived from the equal time of flight argument [14], [20], [35], that is acting on the monomers is 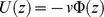, with *ν* the excluded volume parameter. Monodispersity of the brush implies that the potential is parabolic, one thus has 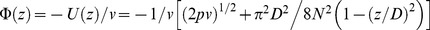. This determines the uncompressed (*p* = 0) brush height 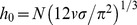 and an intrinsic scale for the pressure 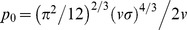. Brushes can thus be compressed to a distance *D* by a pressure 
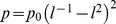
, where *l* = *D*/*h_0_*. The force *F* between two crossed cylinders of radius *R* is given by
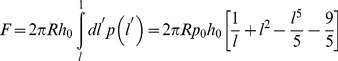
(4)


From Eqn 4, the appropriate normalizing units for the energy per unit surface *F/(2πR)* is the product *p_0_h_0_* and for the distance the normalizing unit is *h_0_*.

As explained in the Results and Discussion section, a modified expression for the MWC monodispersity at higher volume fractions may be obtained by using an EOS 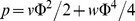. Following above, the potential is then 
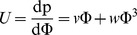
. From the expressions for the EOS and for the potential following from the equal time of flight argument, one obtains 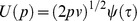, where 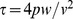 and 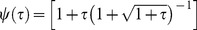. Also, the volume fraction is now given as a function of the potential U by 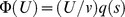, where 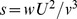 and *q(s)* obeys 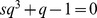. The parabolic nature of the potential and the mass conservation now translate into the implicit distance-pressure expression
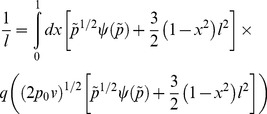
(5)


that can be numerically integrated to provide explicit values of the interaction forces between two crossed cylinders of radius R. Note that all quantities are rescaled as before by the monodisperse MWC result for the brush height *h_0_* and pressure *p_0_*, with 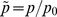 and 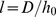.

All distances and energies can be normalized by the monodisperse values of length and pressure. Since 
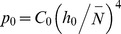
, this allows one to plot all theoretical, numerical, and experimental results in a single plot, where *C_0_* is not a fitting parameter but is given by *2πR*. In addition, we note that the unperturbed height of the polydisperse brush h_0_^*^ may be described empirically by the expression 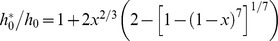, where *x* is (PDI - 1), with 1% accuracy, up to PDI =  2.

### Equation of State

**Figure 1:**
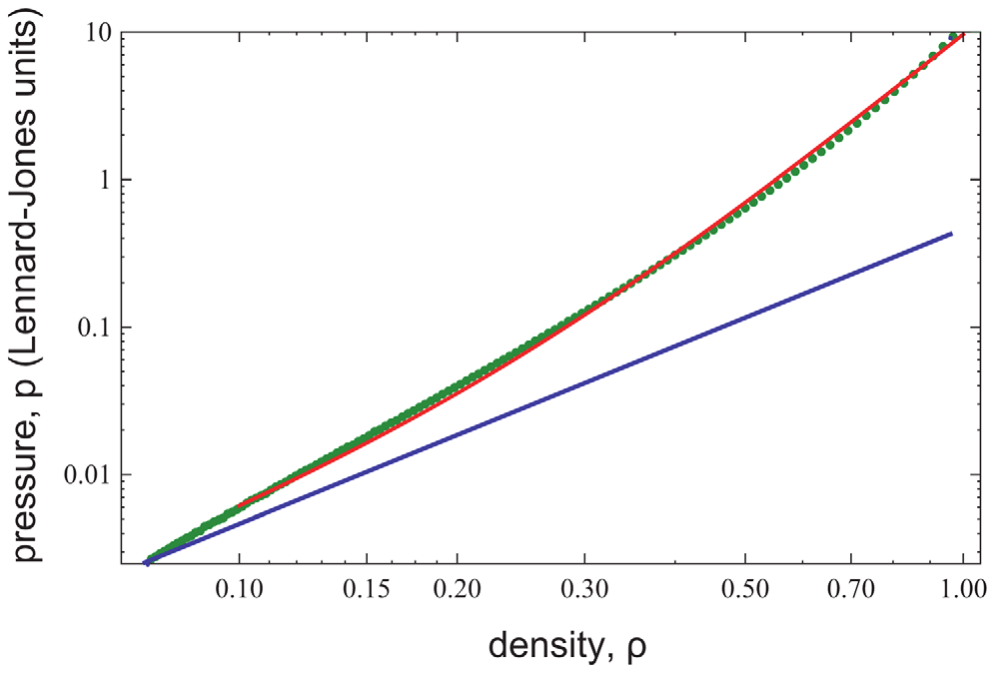
Data from computer simulations of the equation of state (EOS), in Lennard-Jones units (green points), versus a quadratic form of the equation of state (blue line). The green data points represent the EOS of the experimental system. The computer simulations can be accurately modeled by a quartic EOS, 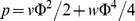, with *v* = 1.0 and *w* = 36.5 to an accuracy better than 10% (red line).

Figure 1 plots computer simulation results (green dots) of the pressure as a function of polymer density, in Lennard-Jones units, corresponding to bulk polymer solution conditions [35]. As for the grafted chains, purely repulsive monomer-monomer interactions were used, together with a potential that mimics the connectivity between adjacent monomers. We considered monodisperse chains with length N = 16. For comparison, a quadratic equation of state (blue line) is shown (classical MWC dilute approximation) and a fitted quartic approximation 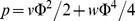 with *v* = 1.0 and *w* = 36.5 (red curve). A quartic form accurately and sufficiently describes the equation of state for the simulation over normalized densities of 0.1 to 1, with a goodness of fit, R^2^ = 0.999. An equation of state with a cubic term, 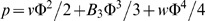, was also used to fit the data and an equal goodness of fit to the quartic form was obtained, R^2^ = 0.999, with a slightly less good reproduction of data for densities from 0.5 to 0.1. Given that the quartic fit was statistically equal to the cubic fit and the quartic form is more computationally tractable, we have chosen the quartic fit for the EOS. The obtained values of *ν* and *w* are based upon the molecular parameters in the simulation, chosen to best approximate the behaviour of polystyrene in toluene, a frequently used model experimental system that is representative of a polymer in good solvent conditions.

## Results and Discussion

**Figure 2:**
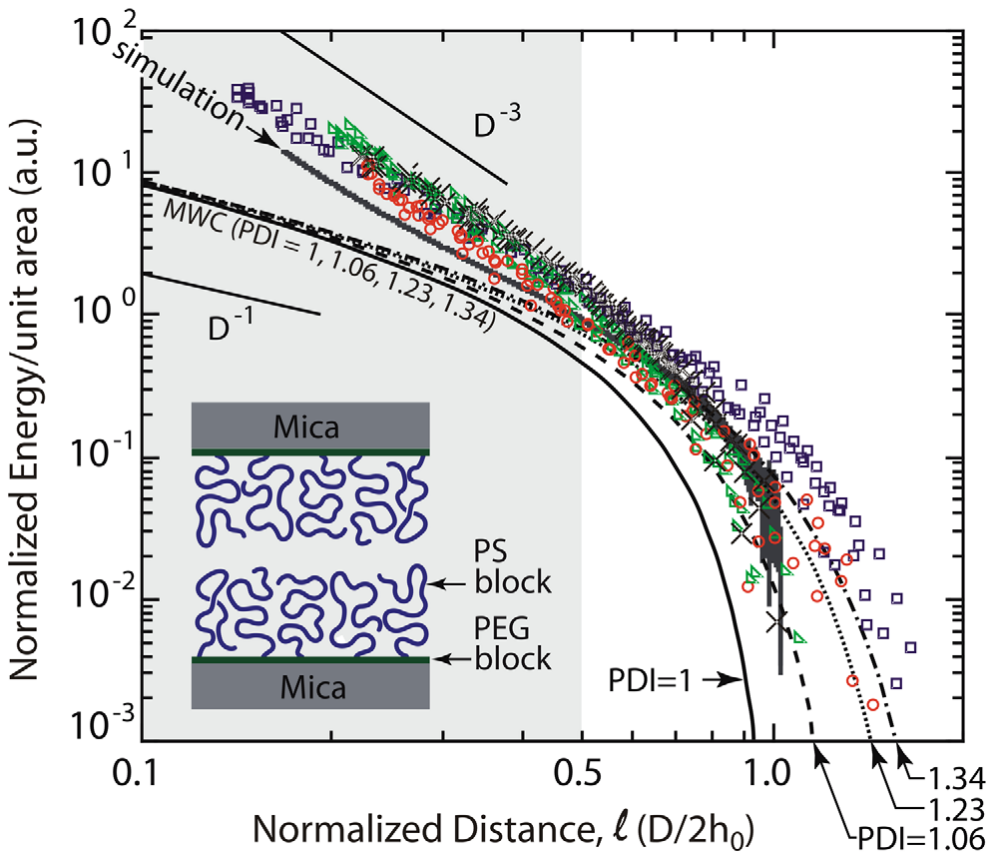
Normalized energy per unit area profiles for the interacting brushes. Experiments were performed with two polydisperse samples PS37k (circles), PS88k (squares), and with a low polydispersity sample PS37k** (triangles), see Table 1. The data are compared to MWC theory for a monodisperse brush (solid black line) and for polydisperse brushes with PDI values of 1.06, 1.23 and 1.34. Computer simulations for the brushes are shown in black. By the MWC theory, *h_0_* is 260 Å for PS37k and *h_0_* is 435 Å for PS88k. The computer simulation parameters were chosen such that the same stretching was achieved as for the PS37k polymer (see Materials and Methods). For comparison, data from Liao and Kuhl [39], for P2VP-PS brushes in toluene are presented (black ‘X’ symbols), with the dimensionless overlap density, 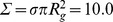, where *σ* is the polymer grafting density and *R_g_* is the radius of gyration of the polystyrene block. The distance is normalized by the experimentally measured brush height, *h_exp_*.

Figure 2 compares simulated and experimentally measured force profiles of two brush-bearing surfaces to the Milner-Witten-Cates (MWC) prediction [20] for the three polymer systems studied (see Materials and Methods for data normalization). We find excellent agreement between experiments and simulation and we note that for the simulations, we cannot present data down to normalized distances of 0.1 as in the experiment. Already for 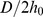 ∼0.2, the simulated brushes are so strongly compressed that the hard core repulsion of the coarse grained monomers becomes apparent. Further compression of the brushes leads to very strong repulsive forces and, finally, to numerical instability. However, despite these limitations, the majority of the experimental compression regime is reproduced by the simulations remarkably. The theory including polydispersity does well in predicting the onset of repulsion and the magnitude of repulsion up to a normalized distance 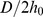 ∼0.5. At higher compressions, both the experimental and the numerical data deviate from the classical MWC theory, regardless of the chain polydispersity, and demonstrate a much stronger decay with distance at a power of −3. Previous work has also noticed that some high density, polymer brush systems, including polystyrene in toluene, exhibit a higher power law at higher compressions [1], [36]–[38], but a validated, quantitative model for this behavior has not been reported to date.

**Figure 3:**
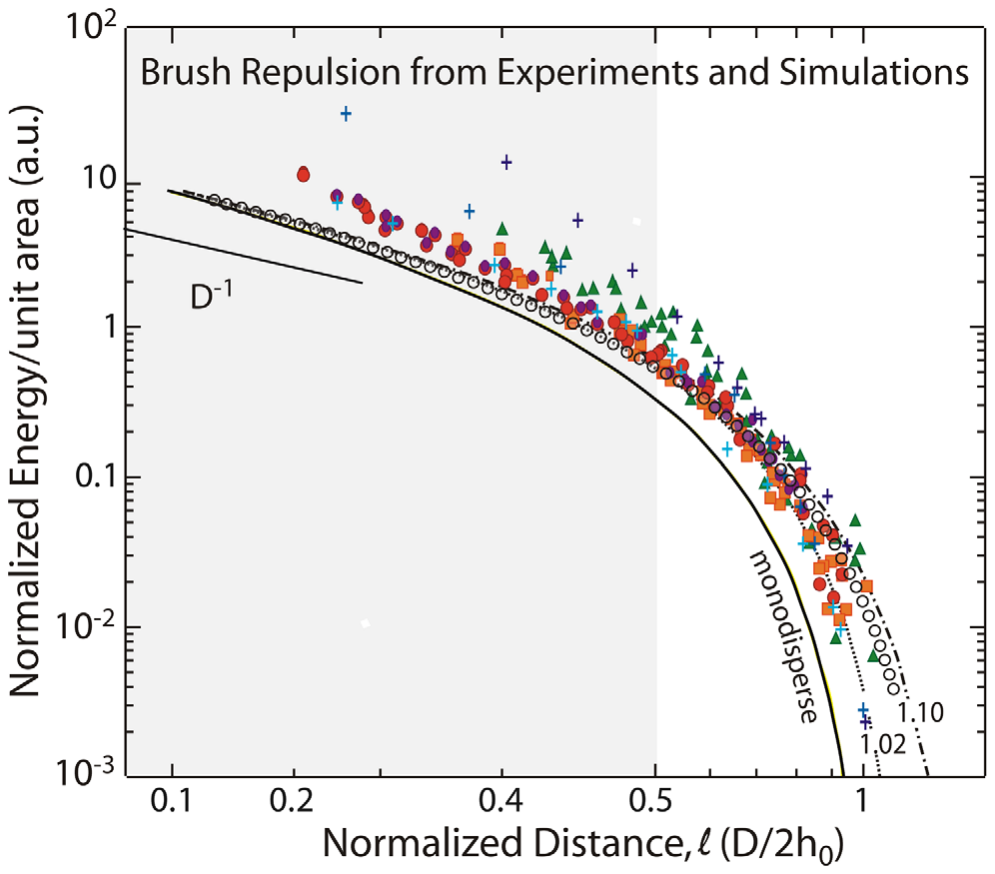
Normalized energy per unit area profiles for two interacting brushes extracted from published data. Filled symbols refer to experimental data points on polystyrene brushes [5], [6]. Open circles are from self-consistent mean field theory [12], [13]. Crosses are from molecular dynamics simulations [19]. Dashed lines were computed from MWC [1], [14], [20] theory for polydisperse brushes with PDI values of 1.02 and 1.10. The MWC prediction for the compression of monodisperse brushes does not fit the data. Including polydispersity in MWC accounts for the lower compression regime, but misses by one order of magnitude the deviations at high compressions, commencing at 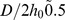 (shaded region), that have never been addressed quantitatively by theory (see Materials and Methods for details on the normalization). For SFA data from refs 5 and 6, the exact *R* for each experimental data set could not be derived from the published papers. Under these conditions, *C_0_* becomes an arbitrary scaling factor for the y-axis (see Data Normalization under Materials and Methods).

From a literature survey, it is clear that previous attempts to accurately compare theory or simulations to experiments have met two major difficulties. The first difficulty is the strong influence of sample polydispersity on the onset of the repulsive forces; a moderate polydispersity of 10% is able to shift the onset of the repulsive forces by 50%. This has been readily dealt with in the literature [1], [9] and is considered here simply to demonstrate full treatment of all compression regimes (Figure 2). The second, more fundamental difficulty is related to the large deviations at strong compressions between the experimentally observed and the predicted compression power laws. When compression increases the brush monomer volume fraction beyond the semi-dilute regime, the quadratic pressure EOS, 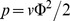, assumed by MWC theory, no longer holds and a more complete relation needs to be used [1], [14], [34]. In both the experiments and simulations of Figure 2, this effect is readily observed by a significant deviation from the MWC monodisperse/polydisperse theory for 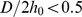 (Figure 2). To further demonstrate this point, Figure 3 illustrates some examples from the literature of brush-brush interaction profiles, where the systems were considered to be in good solvent conditions. These data also exhibit deviations in the high compression regime.

**Figure 4:**
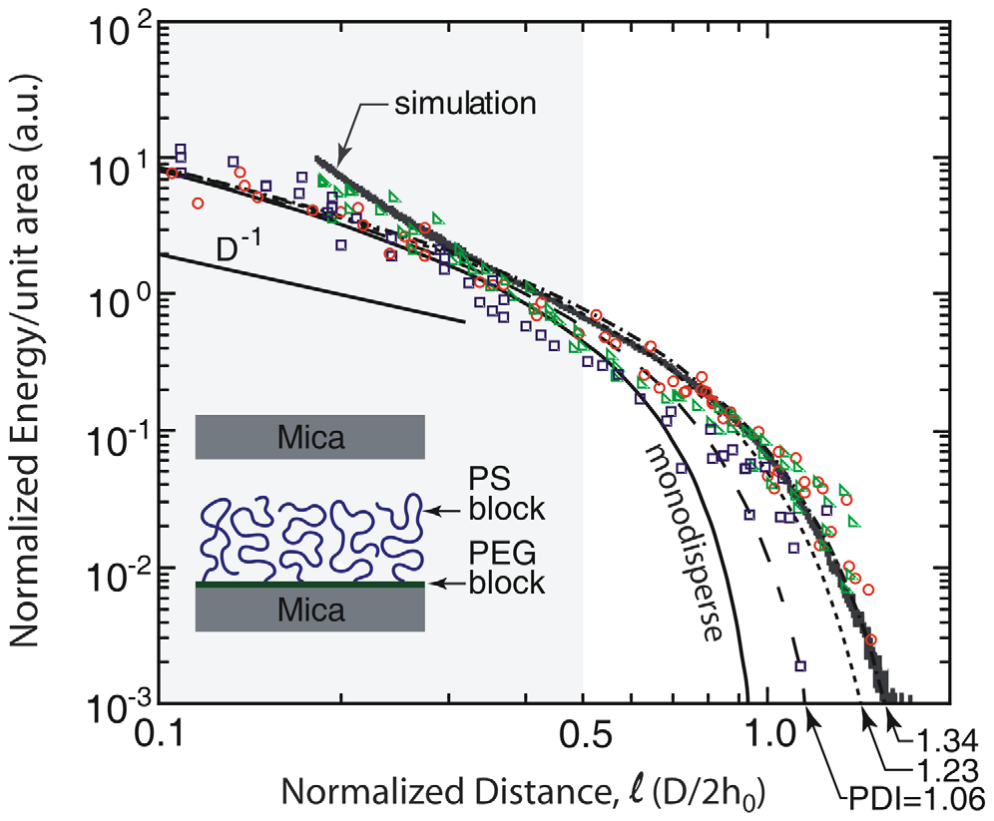
Normalized energy per unit area profiles for a single brush interacting with a hard wall (mica). (see Materials and Methods for details on the normalization). In this case, the grafting density of the brush is not as high as in the double brush case. As a result, the deviations from theory are not apparent.

In addition to double brush experiments, single brush experiments were performed. From Figure 4, it is seen that the agreement between experiment and simulation data covers both the onset of repulsion, i.e., where the repulsion vanishes to zero, and a large range of compression for the three different polymer systems of the lower density case. Here, MWC theory for monodisperse chains shows good agreement with the data at high compressions, D/h_0_<0.5. Since the force normalization is the same for both the single and double brush cases, Figure 2 actually displays forces that are, at equivalently strong compressions, larger by roughly a factor ten than in Figure 4. Previous work has also reported that some high density, polymer brush systems, including polystyrene in toluene, exhibit a higher power law at higher compressions [6], [11], [17]–[19]. It appears that in our experimental system, the single brush system does not exhibit the same high compression behavior as the double brush system. It is likely that our single brush system is at a lower surface density than the double brush system, due to the process of exchanging one of the brush-coated surfaces with a bare mica surface and the subsequent equilibration with a bare mica surface. Some transfer of polymer between the surfaces would result in a lower grafting density than in the double brush system.

The high monomer concentration is the key factor in the high compression deviations seen in the double brush system. This means that in the regime of high monomer density, in the semi-dilute and concentrated regime, higher order interactions become important. However, the question still remains – what higher order interaction terms are necessary to accurately describe the high compression regime when simple pair-wise repulsive interactions and dilute or semi-dilute solution conditions are no longer sufficient?

In order to derive an appropriate EOS for a polymer in good solvent conditions, such as polystyrene in toluene, we obtained computer simulation data [35] of the pressure as a function of concentration for such a system, where monomer-monomer interactions are purely repulsive. This simulated data fit the analytical form of 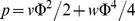 with *v* = 1.0 and *w* = 36.5 to an accuracy better than 10% within the relevant range of the experiments (see Materials and Methods). The absence of a Φ^3^ term in the EOS appears rather surprising. We do not state that this means 3-body interactions are not present. Rather, following our simulation data, the EOS is overall slightly better described by the expression 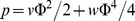. However, we cannot provide a substantiated physical explanation for the absence of the Φ^3^ term in the EOS, just that the inclusion of the cubic term did not substantially improve the fits. Moreover, the inclusion of the cubic term increases the computational load. We have, therefore, chosen to use the more simplified and computationally tractable quartic form. For our purposes here, we do not conclude that there is an absence of 3-body interactions at high compression, but that 3-body interactions do not occur without 4-body interactions.

**Figure 5:**
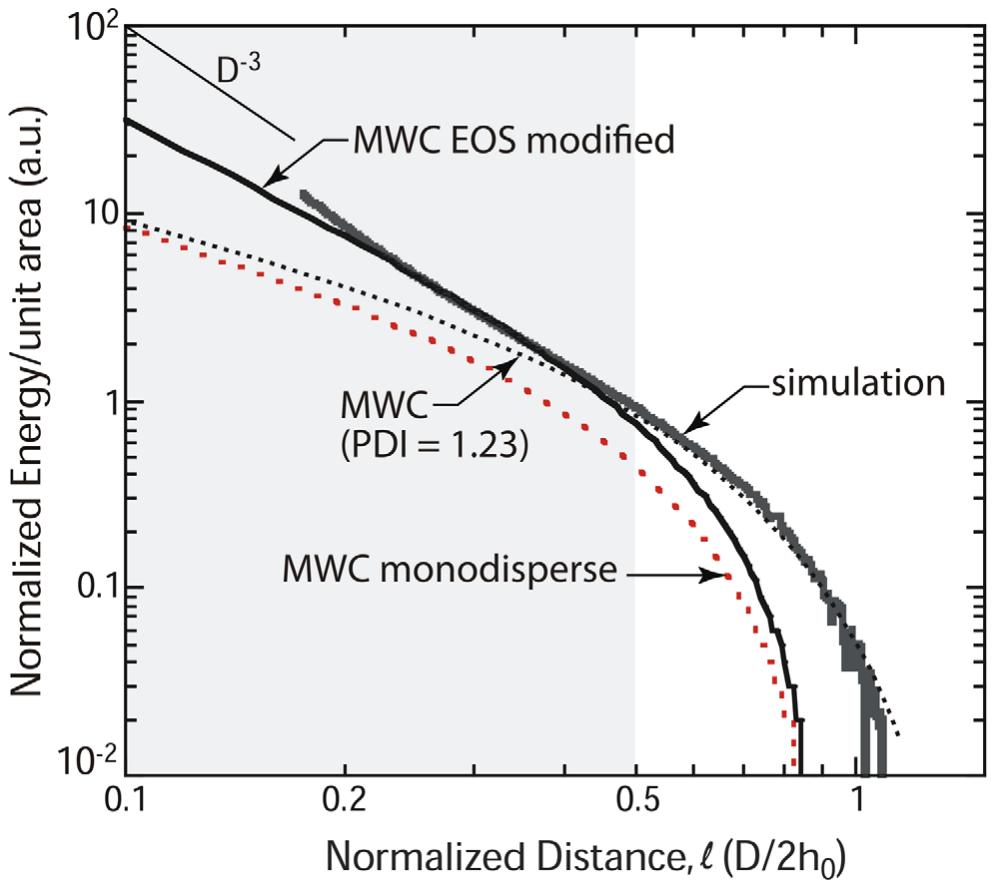
Computer simulations (grey curve) describe quantitatively our brush-brush experiments in **Figure 2** . Here, we compare the energy per unit area as a function of distance of the computer simulations with the monodisperse MWC theory, the polydisperse MWC theory, and our approach with the modified EOS with 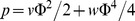. The polydisperse MWC theory describes well the outer, weak compression portion of the curve (Figure 2), while the inner, strong compression limit requires accounting for deviations in the pressure EOS.

Subsequently, a modification of the MWC theory was used to derive the force-distance curves of Figure 5, where the potential 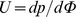 was given by the improved EOS. We also comment that it should be straightforward to apply a similar technique to other polymer systems of interest. By tuning the interactions between monomers in the computer simulation to match the experimental conditions, one should be able to model and obtain the appropriate equation of state for other systems using a generic expression for the pressure: 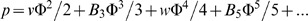, with appropriate coefficients. As shown in Figure 5, the interaction forces obtained from the modified MWC theory with an accurate EOS are in very good agreement with our simulation data and therefore with the experimental data shown in Figure 2. We further note that, with the more accurate EOS, the potential becomes 
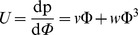
, which yields an 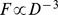 dependence at high compressions (Figure 2). When comparing experiments and simulations to theory, it is not only important to account for the polydispersity at low compressions, but it is also essential to consider higher order interactions, via a modified EOS. Our results demonstrate that it can be critical to account for the monomer density increase that can occur with confinement of sufficiently dense polymer brushes in the 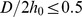 regime.

One might be tempted to propose that, in the high compression regime, there may be a change in solvent quality and hence, the excluded volume parameter, as previously proposed for P2VP-PS grafted brushes at high density [39]. First, we note that the work of Liao and Kuhl did not consider if an improved EOS would better fit their data. One representative data set from ref 39, with dimensionless overlap surface density Σ = 10.0, is included in Figure 2 (black ‘X’ symbols). We find that this data set for P2VP-PS brushes agrees well with our experimental data for PEG-PS brushes and a modified EOS may be an alternate explanation for the deviations observed in their P2VP-PS brushes. Second, changes in solvent quality cannot appear in our simulation model, where the solvent is treated implicitly and good solvent quality is assured via a purely repulsive excluded volume interaction (athermal solvent). As this modified EOS also is capable of describing our simulation data of polymer brushes in Figure 5, and the brush simulation data agrees well, to a large extent, with our experimental data, and that of ref 39 (Figure 2), we assume that a change of solvent quality upon compression is a marginal effect in our experiments.

Under conditions of high compression, both a single polymer brush and a double polymer brush are strongly confined. As noted, the double brush system requires the introduction of higher order terms in the EOS to account for higher order interactions (Figure 5) and thus more accurately describe the interaction energy at smaller normalized distances, D/2h_0_<0.5. It is in this region, where the brush interpenetration may become important in a double brush system, as at D/h_0_∼0.5 the brushes are beginning to come into contact. Although interpenetration is not allowed in MWC theory and our experiments cannot distinguish between the effects of brush confinement and interpenetration, interpenetration of opposing polymer chains has been previously reported [40], [41]. Regardless, the net effect of compressing the brush is a sharp increase in the local monomer concentration, with a concomitant increase in osmotic pressure, and hence a marked increase in the normal load.

In developing a more accurate EOS for such polymer systems, our results suggest that it is not critical to determine whether the increase of monomer concentration is strictly due to simple polymer confinement or interpenetration. Rather, the appropriate EOS properly accounts for changes in the osmotic pressure with concentration and thus yields the appropriate response in normal load. Although the force profile may be insensitive to interpenetration, it must be noted that chain interpenetration in opposing polymer brushes has significant impact on the lubrication properties between the brushes, as was previously shown in experiments [36] and more recently demonstrated in computer simulations by Galuschko et al. [42] and Spirin et al. [43]–[45].

## Summary

In summary, force profiles were obtained for both single and double PEG-PS copolymer brush systems, selectively adsorbed onto mica surfaces in toluene. For single brushes, the chain polydispersity significantly shifts the onset of repulsion. However, the polydispersity-corrected MWC theory, which is based on a quadratic equation of state appropriate for dilute solutions, accurately predicts the measured force profiles for these systems, as the monomer volume fractions are relatively small. As a result, the predicted power law of 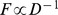
at high compressions can be observed only with sparse brush systems.

The force profiles obtained from the high grafting density double brush systems also are well captured by the polydispersity-corrected MWC theory at low concentrations. At high compressions, we find the impact of chain polydispersity is modest. However, the measured repulsion exceeds MWC predictions by one order of magnitude. In this regime, where monomer volume fractions are larger high, both the SFA experimental data and the simulation data for the same geometry agreed remarkably, giving an exponent of −3 at normalized distances less than 0.5.

Our analysis shows that the order of magnitude difference in the forces is a result of deviations from ideality in the constitutive equation of state for bulk polymer solutions. We have shown that such behavior can be quantitatively modeled if one uses an appropriate pressure equation of state. The EOS used in our calculations was measured independently from computer simulations and extends the conventional quadratic pressure dependence on volume fraction to higher concentrations. In addition, this model may be applied to other polymer systems and we suggest that this behavior is indicative of the universal behavior of polymers in good solvent conditions. We further comment that the brushes studied here can withstand remarkably high loads while retaining a liquid structure and reversible elasticity. Related computer simulations [42]–[45] at comparable high loads also show that such layers still maintain their lubrication properties.

Significantly, these findings are expected to apply quite generally beyond the specific case of end-grafted non-ionic polymers studied here. Indeed, at high compressions, one expects universal compression behavior - dictated only by the structure of the confined polymer liquid and independent of the polymer attachment method to the surface. In the case of ionic polymer systems, the contribution of counter ions to the osmotic pressure becomes subdominant at such high polymer concentrations. Thus, contrary to the low-pressure limit [46], we expect our predictions at high loads to hold in general for the vast majority of interfacial polymer layers including polyelectrolytes, for which charges are known from simulations to modestly change the brush lubrication properties [47].
